# Pituitary apoplexy presented with optic neuritis

**Published:** 2013

**Authors:** Abdorreza Naser Moghadasi, Razieh Aghakhani, Mahsa Owji, Mansoureh Togha

**Affiliations:** 1 Sina MS Research Center, Sina Hospital, Tehran University of Medical Sciences, Tehran, Iran; 2 Public Health and Sustainable Development Center, North Khorasan University of Medical Sciences, Shirvan, Iran; 3 Department of Neurology AND Sina Hospital, Tehran University of Medical Sciences, Tehran, Iran

**Keywords:** Pituitary Apoplexy, Optic Neuritis, Brain MRI

The patient is a 40-years-old woman presented with visual loss in the right eye since two days ago. The patient complained of headache with gradual onset in the right parietal area since 2 years ago. The headache pattern did not alter.

The left eye was normal but the vision in the right eye was 50 cm finger counting. Marcus Gunn pupil could be observed during swinging-flashlight test in the right eye. According to these findings, the first diagnosis was optic neuritis which was corroborated with visual evoked potential (the latency of P100 was 122). According to magnetic resonance imaging (MRI), there was a big cystic tumor in sella turcica extended to the suprasellar region (Figure 1-A). It had a large liquid component. Optic chiasm was under pressure and was displaced (Figure 1-B). A faint enhancement was seen after the injection (Figure 1-C).

**Figure 1:**
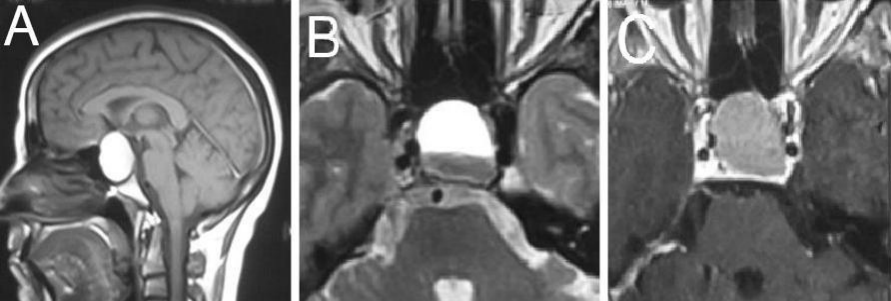
A- In sagittal T2's view, a big cystic tumor was seen in sella turcica that was extended to the suprasellar region; B- In coronal T2's view, it can be seen that optic chiasm was displaced and right optic nerve was under pressure by this cystic mass; C- MRI with gadolinium showed a faint enhancement around the tumor

The patient was operated with the transnasal-transsphenoidal approach. Blood and fibrin were among the small monomorphic round cells and fibro-connective tissue of the pituitary gland. Considering the bleeding inside the pituitary tumor, the final diagnosis was pituitary apoplexy. After recovery from the operation the symptoms were removed.

Pituitary apoplexy presents with headache, loss of vision, ophthalmoplegia, and mental alterations which is caused by infarction or sudden bleeding inside the pituitary tumor.^1^

In our review of literature, 3 patients afflicted with pituitary apoplexy presented with optic neuritis were reported.

In a study conducted by Petersen et al., all three patients referred to them suffered from unilateral visual loss and headache. They were examined with the early diagnosis of optic neuritis. After taking a brain computerized tomography (CT) scan, pituitary adenoma together with the formation of cyst were detected in patients. After transsphenoidal adenomectomy, the patient regained full vision. Therefore, the correct diagnosis of these patients, who had been incorrectly diagnosed with optic neuritis, was pituitary apoplexy.^2^

## References

[1] Ranabir S, Baruah MP (2011). Pituitary apoplexy. Indian J Endocrinol Metab.

[2] Petersen P, Christiansen KH, Lindholm J (1988). Acute monocular disturbances mimicking optic neuritis in pituitary apoplexy. Acta Neurol Scand.

